# Serological Study of Fascioliasis Using Indirect ELISA in Gorgan City, Golestan Province, Northern Iran

**DOI:** 10.18502/ijpa.v15i3.4207

**Published:** 2020

**Authors:** Negar BAHRAM, Mitra SHARBATKHORI, Farideh TOHIDI, Reza GHASEMIKHAH

**Affiliations:** 1. Student Research Committee, Arak University of Medical Sciences, Arak, Iran; 2. Infectious Diseases Research Center, Golestan University of Medical Sciences, Gorgan, Iran; 3. Department of Parasitology & Mycology, School of Medicine, Golestan University of Medical Sciences, Gorgan, Iran; 4. Laboratory Sciences Research Center, Golestan University of Medical Sciences, Gorgan, Iran; 5. Infectious Diseases Research Center (IDRC), Arak University of Medical Sciences, Arak, Iran; 6. Department of Parasitology & Mycology, School of Medicine, Arak University of Medical Sciences, Arak, Iran

**Keywords:** Seroprevalence, Human fascioliasis, Serodiagnosis, Iran

## Abstract

**Background:**

Fascioliasis is a neglected zoonotic disease, caused by *Fasciola* species in human and livestock. We aimed to detect the seroprevalence of human fascioliasis Gorgan City, Golestan Province, northern Iran using ELISA method in 2017.

**Methods:**

Overall, 612 serum samples were analyzed. A relevant questionnaire for demographic data was obtained for all cases. An indirect ELISA test was used to detect IgG antibodies against *Fasciola* in the sera. The data analysis was performed employing SPSS program version 21.

**Results:**

Eleven cases (1.79%) were seropositive for fascioliasis. The seroprevalence of fascioliasis was 1.9% and 1.1% among males and females, respectively. There was no statistically significant association between the fascioliasis and analyzed variables such as sex, age, residence, job, education, etc.

**Conclusion:**

This study was conducted only on the people referring to the Reference Laboratory of Gorgan. It cannot be distributed to the whole city. Thus, due to importance of the disease, finding the seroprevalence of fascioliasis in a comprehensive survey in Golestan Province should be accounted in further studies.

## Introduction

Fascioliasis or fasciolosis, caused by two species of liver flukes *Fasciola hepatica* and *F. gigantica*, is one of the major neglected zoonotic diseases in the world especially in the Middle East and some African countries (1). Human fascioliasis is considered as a food and water borne disease (2). The disease is primarily an infection of ruminants such as sheep and cattle and humans role as accidental hosts via consuming some fresh aquatic vegetables such as water-cress or drinking water contaminated to meta-cercariae of *Fasciola* spp. (3).

*Fasciola* generally is stayed in biliary ducts or gall bladder of the definite host but occasionally might be observed in ectopic places like skin, eye, spleen, etc. (1). Snails of Lymnaeidae family are role as intermediate host of *F. hepatica* and *F. gigantica* (4).

Fascioliasis is speared out where livestock such as sheep and cattle are fostered and served as important reservoirs for the parasite, causing economic loss arising from wasting infected organs, decreasing milk, meat, and wool productions, decreasing weight and fertility (1). Furthermore, fascioliasis is an important zoonotic issue in public health with an estimate of 2.4–17 million people to be infected in Asia and Africa and 91.1×10^6^ people at risk of infection (5). Infected livestock are a major source of human *Fasciola* infection since grazing areas of these animals is generally close to the freshwater plants (4).

The infection rates of 0.1% to 91.4% has been reported in various livestock of Iran. The country has a history of two human outbreaks. The first outbreak of human fascioliasis, involved about 10000 people in Guilan Province, northern Iran, in 1989. The second outbreak happened about 10 yr later that involved more than 10000 people in the same province (6), and it is believed to be the biggest fascioliasis outbreak in 20^th^ century (7).

Currently, coprological techniques, serological tests and other non-invasive diagnostic methods such as ultrasound, radiology, computed tomography or magnetic resonance are employed for diagnosis of human fascioliasis. Serological tests are useful in all stages of the disease and especially during the acute phase (5). Anti-*Fasciola* antibodies can be identified in the serum even two weeks after the infection. They have 100% and 97.8% sensitivity and specificity to detect the fascioliasis, respectively (8).

There is no previous study on fascioliasis in the Gorgan area. Regarding to high medical and economic importance of the disease, this study was conducted to determine the sero-prevalence of human fascioliasis in people referring to Reference laboratory of Gorgan, Golestan Province, northern Iran using indirect ELISA procedure.

## Materials and Methods

### Samples

The serum samples were obtained from another descriptive cross sectional study on hydatidosis (9), from people attending to Reference Laboratory of Golestan University of Medical sciences, Gorgan City, capital of Golestan Province, northern Iran in 2017. Three milliliter of venous blood sample was obtained from each person and sera were kept at −20 °C. Then sera transferred in cold conditions to the Parasitology Laboratory of Tehran University of Medical Sciences, Tehran, Iran for further analysis.

The study was approved by Ethics Committee of the Arak University of Medical Sciences with confirmation No. IR.ARAKMU.REC.1397.104.

A detailed demographic data and relevant history had been recorded in questionnaires such as age, gender, residency (urban/rural), tribes (Fars, Sistani, Turkmen, etc.), literacy, occupation, history of eating raw vegetables, proceeded olive (Persian name: Zeitoon parvardeh). The stored sera at −20 °C, were transferred in cold conditions to the parasitology laboratory of Tehran University of Medical Sciences for consequent analysis.

### Antigen preparation

Infected livers to *Fasciola* were obtained from Tehran abattoirs and transferred to the laboratory of Helminthology, School of Public Health, Tehran University of Medical Sciences, Tehran, Iran. *F. hepatica* and *F. gigantica* parasites were separated from liver, and washed 6 times with serum physiology. Then were homogenized in 0.045 mM PBS/pH 7.2 employing electrical homogenizer. Afterward, sonication was performed and centrifuged in 10000 g at 4 °C for 15 min (10). The supernatant was stored in refrigerator for later analysis.

### Indirect Enzyme Linked Immunosorbent Assay (ELISA)

The ELISA was performed based on the method already established (11). Positive and negative control samples were obtained from confirmed fascioliasis patients and 30 healthy people in non-endemic regions without history of fascioliasis, respectively. OD was measured using an ELISA reader (BioTek, USA) at 490 nm. The cut-off point was considered as 3SD above the mean of controls.

### Data statistical analysis

To detect the relationship between variables and risk of human fascioliasis, odds ratio (OR) and 95% confidence interval (CI) were estimated using logistic regression model. *P* value less than 0.05 was accounted significant. The data were analyzed employing SPSS (Chicago, IL, USA) (Statistical Package for Social Science) program version 21.

## Results

Among 612 blood samples, 86 (14.1%) and 526 (85.9%) were male and female, respectively. The subjects were between 6 to 80 yr old with a mean age of 41.3±14.7. Cut-off was obtained as 0.22 and each OD absorbance higher than this rate was calculated as positive. Eleven (1.79%) people including 1 male and 10 females were positive for fascioliasis. Although females (1.9%) indicated a higher prevalence than males (1.16%), the difference was no statistically significant.

The infection rate was 1.24% and 2% in age group below/equal and above 40 yr old, respectively. There was not any significant relationship between the age and the disease. The infection rate in Fars and non-Fars were as 1.58% and 2.35%, respectively. There was no significant correlation between the tribes and the fascioliasis. Among 11 seropositive, 7 and 4 cases were urban and rural, respectively. Data analysis did not indicate any significant association regarding the residence. In terms of occupation, 9 (81.8%) and 2 (18.2%) seropositive subjects were housekeepers and employees, respectively. No statistical significant differences were obtained among different jobs. 1.37% and 3.92% of educated and non-educated people were infected to fascioliasis, respectively. There was no significant relationship between the fascioliasis and level of literacy. In addition, there was no significant association between the consuming raw vegetables or olive crushed with the disease (Table 1).

**Table 1:** Logistic regression analysis of seropositive cases of fascioliasis according to studied variables in people referring to Reference laboratory of Gorgan

***Variables***		***Seroprevalence***					
		***No. of Positive (%)***	***No. of Examined (%)***	***Odds Ratio (95% CI^a^)***	***P-value***
Sex	Male	1 (9.1)	86 (14.1)	Reference^b^	0.9
	Female	10 (90.9)	526 (85.9)	0.931(0.064–13.64)	
Age (yr)	≤ 40	5 (45.5)	313 (51.1)	Reference	0.7
	> 40	6 (54.5)	299 (49.9)	1.26(0.29–5.4)	
Tribes	Fars	7 (63)	442 (72.2)	Reference	0.6
	Non-Fars	4 (37)	170 (27.8)	0.739(0.24–2.67)	
Residence	Urban	7 (63.6)	409 (66.8)	Reference	0.9
	Rural	4 (36.4)	203(33.2)	1.07(0.029–3.94)	
Occupation	Housekeeper	9 (81.8)	425 (69.4)	Reference	0.567
	Employee	2 (18.2)	39 (5.2)	4.11(0.7–23.8)	
	Farmer	0 (0)	26 (4.2)		
	Other	0 (0)	129 (21.2)		
Education	Educated^c^	7 (63.6)	510 (83.3)	Reference	0.12
	Non-educated	4 (36.4)	102 (16.7)	2.93 (0.8, 10.9)	
Use of raw vegetables	Yes	10 (90.9)	506 (82.7)	Reference	0.3
	No	1 (9.1)	106 (17.3)	2.58(0.3–21.2)	
Use of olive crushed	Yes	3 (27.3)	205 (33.5)	Reference	0.7
	No	8 (72.7)	407 (66.5)	0.7(0.19–3.1)	

a CI: confidence interval

b Reference: The level of variable which other levels compared with that

c Educated: defines as primary, secondary school, high school and university

## Discussion

A seropositivity of 1.79% was obtained for fascioliasis in persons referring to Reference Laboratory of Gorgan, employing indirect ELISA test.

According to literature livestock fascioliasis is endemic in different parts of Iran. Although, the higher infection rates of the disease were seen in livestock of southern parts, the human fascioliasis has been mostly reported from northern parts especially in Guilan Province (6).

The reported seroprevalence (1.79%) in this study, is in agreement with infection rates were reported from different regions in Iran such as Isfahan (1.7%) (12), Yasuj (1.86%) (13), Meshkin-Shahr (1.96%) (14), Guilan (1.36%) (15), Lorestan (1.3%) (16).

However, there are some regions with less prevalence (hypoendemic) in the country e.g. Chaharmahal and Bakhtiari (0.135%) (17), Kermanshah (0.5%) (4), Ilam (0.66%) (18), and Pirabad (0.7%) (19). In this study, the infection rate in women (1.9%) was more than men (1.16%) that is in conflict with some studies which observed a higher infection rate in men and reported 28.3% Vs. 21% and 2.83% Vs. 1.3% seropositivity in men and women, respectively (10,13). However, our study is in agreement with studies in Kermanshah (4), Ilam (18), Lorestan (16), Isfahan (12) Guilan (15), in the country and also Turkey (20, 21), Mexico (22) and Peru (23) that reported higher infection rates in women than men. This may be due to more cooking activities of women e.g. cleaning and washing vegetables, catering salads, etc. This research was not in concordance with a study in Isfahan district (12) within the country, Turkey (20), Andean countries and the Egypt (24) which indicated a significant correlation between the sex and fascioliasis but was in concordance with many studies that were not reported such a significant difference in Iran (4, 14, 15, 18, 19), Turkey (21) and Bolivia (25).

In the current study, seropositivity of fascioliasis was higher in people above 40 yr old (2%) than younger individuals (1.24%) that is in concordance with general age pattern of human fascioliasis in Iran harboring adults more than children (14). Studies in Meshkin-Shahr (26), Yasuj (13), Guilan (15), Isfahan (12), indicated the most seropositivity of the disease in 40–49, 41–50, 40–59 and more than 60 yr old, respectively. The association between age and fascioliasis was not significant in our study that was in agreement with other studies in the country (4, 13–16, 26), Turkey (20) and Africa (24).

No significant correlation was observed between different tribes and fascioliasis in our study. The seropositivity in Fars people was less than non-Fars people (1.58% vs. 2.35%).

Considering the living area, rural (1.97%) and urban (1.71%) life showed no significant relationship with the disease. That is in concordance with studies performed in Guilan (15) or Isfahan (12) and in disagreement with the study of Lorestan, which indicated a significant difference between rural (1.9%) and urban (0.4%) area with fascioliasis (16). Although the infection rate of urban (2.4%) was less than rural (1.4%) subjects in Meshkin-Shahr, the difference was not significant (14).

Farmers have a greater risk of infection to *fasciola* because of close contact with livestock reservoirs, snails, aquatic plants and unsafe water. However, all of 26 farmers in our study were seronegative. In return, employees (5.12%) followed by housekeepers (2.11%) indicated the highest seropositivity. It could be result of low number of farmers (26 of 612) compare with other jobs in the study (Table 1). In a study from Meshkin-Shahr unemployment cases had the highest rate (5.9%) of infection (14).

Non-educated cases indicated more *Fasciola* seropositivity (3.92%) than educated cases (1.37%). Literacy did not show any significant correlation in our study. That is in agreement with previous studies in Guilan (15), Meshkin-Shahr (14), Kermanshah (4), Ilam (18), Lorestan (16) and Isfahan (12).

People using raw vegetables were more infected to fascioliasis (1.97% vs. 0.94%) with no significant difference that is in agreement with reports from Meshkin-Shahr (14) and Yasuj (13). Consuming fresh local aquatic vegetables have been reported as a significant factor for the disease in some studies in the country (16, 19). Dalar and proceeded olive (Zeitoon parvardeh) are two important sources of fascioliasis in the northern Iran (27). In the current study, people consuming olive crushed were less infected to fascioliasis (1.46% vs. 1.96%) with no significant difference.

Overall, due to relatively high seropositivity (1.79%) of fascioliasis among people referring to the Reference Laboratory of Gorgan, future researches involving more cases from the city and Golestan province is recommended. In addition, public health education conducting preventive criteria should be applied by the authorities.

## Conclusion

Infection rate of human fascioliasis (1.79%) was observed in the present research. The results cannot be generalized to the province. Thus, due to economic and public health importance of the disease, conducting a comprehensive research on both serological and coprological finding of fascioliasis in the Province may be accounted for future investigations.

## Ethical considerations

Ethical issues (Including plagiarism, informed consent, misconduct, data fabrication and/or falsification, double publication and/or submission, redundancy, etc.) have been completely observed by the authors.

## References

[1] Rokni MB Lotfy WM Ashrafi K Fasciolosis in the MENA Region in: McDowell MA Rafati S, editors. Neglected Tropical Diseases-Middle East and North Africa. Vienna: Springer; 2014 p. 59–90.

[2] Ashrafi K Bargues MD O’Neill S Fascioliasis: a worldwide parasitic disease of importance in travel medicine. Travel Med Infect Dis. 2014; 12(6):636–49. 25287722 10.1016/j.tmaid.2014.09.006

[3] Rokni M. Helminth-Trematode: *Fasciola hepatica* and *F. gigantica*. In: Motarjemi Y, editor. Encyclopedia of Food Safety. 1st ed Amsterdam: Elsevier/Academic Press; 2015 p. 140–5.

[4] Bozorgomid A Nazari N Rokni MB Epidemiology of fascioliasis in Kermanshah Province, western Iran. Iran J Public Health. 2018; 47(7):967–972. 30181994 PMC6119572

[5] Mas-Coma S Valero MA Bargues MD. Fascioliasis, in Digenetic Trematodes, Toledo R Fried B, Editors., Springer International Publishing: Cham; 2019 p. 71–103.

[6] Ashrafi K. The status of human and animal fascioliasis in Iran: A narrative review article. Iran J Parasitol. 2015; 10(3):306–328. 26622287 PMC4662732

[7] Saebi E Parasitic Diseases in Iran (in Persian). Tehran, Iran: Ayeezh; 2009.

[8] Rokni MB Bozorgomid A Heydarian P Molecular Evidence of Human Fasciolosis Due to Fasciola gigantica in Iran: A Case Report. Iran J Public Health. 2018; 47(5):750–4. 29922619 PMC6005974

[9] Fathi S Ghasemikhah R Mohammadi R Seroprevalence of hydatidosis in people referring to Reference laboratory of Gorgan, Golestan Province, northern Iran 2017. Iran J Parasitol. 2019; 14(3):436–443. 31673262 PMC6815868

[10] Aryaeipour M Eshrat Beigom K Heidari Z Serological study of human fasciolosis in patients referring to the School of Public Health, Tehran University of Medical Sciences, Tehran, Iran during 2008–2014. Iran J Parasitol. 2015; 10(4):517–22. 26811716 PMC4724826

[11] Rokni MB Massoud J O’Neill SM Diagnosis of human fasciolosis in the Gilan province of Northern Iran: application of cathepsin L-ELISA. Diagn Microbiol Infect Dis. 2002; 44(2):175–9. 12458125 10.1016/s0732-8893(02)00431-5

[12] Saberinasab M Mohebali M Molawi G Seroprevalence of human fascioliasis using indirect ELISA in Isfahan district, central Iran in 2013. Iran J Parasitol. 2014; 9(4):461–5. 25759726 PMC4345084

[13] Sarkari B Ghobakhloo N Moshfea A Seroprevalence of human fasciolosis in a new-emerging focus of fasciolosis in Yasuj district, southwest of Iran. Iran J Parasitol. 2012; 7(2):15–20. 23109941 PMC3469183

[14] Asadian S Mohebali M Mahoudi M Seroprevalence of human fascioliasis in Meshkin-Shahr district, Ardabil Province, northwestern Iran in 2012. Iran J Parasitol. 2013; 8(4):516–21. 25516731 PMC4266114

[15] Ashrafi K Saadat F O’Neill S The endemicity of human fascioliasis in Guilan province, northern Iran: the baseline for implementation of control strategies. Iran J Public Health. 2015; 44(4):501–11. 26056669 PMC4441963

[16] Heydarian P Ashrafi K Mohebali M Seroprevalence of human fasciolosis in Lorestan Province, western Iran, in 2015–16. Iran J Parasitol. 2017; 12(3):389–97. 28979349 PMC5623919

[17] Manouchehri Naeini K Mohammad Nasiri F Rokni MB Seroprevalence of human fascioliasis in Chaharmahal and Bakhtiyari Province, Southwestern Iran. Iran J Public Health. 2016; 45(6):774–80. 27648421 PMC5026833

[18] Abdi J Naserifar R Rostami Nejad M New features of fascioliasis in human and animal infections in Ilam province, Western Iran. Gastroenterol Hepatol Bed Bench. 2013; 6(3):152–5. 24834263 PMC4017511

[19] Kheirandish F Kayedi MH Ezatpour B Seroprevalence of human fasciolosis in Pirabad, Lorestan Province, Western Iran. Iran J Parasitol. 2016; 11(1):24–9. 27095965 PMC4835466

[20] Cengiz ZT Yilmaz H Dulger AC Seroprevalence of human fascioliasis in Van province, Turkey. Turk J Gastroenterol. 2015;26(3)259–62. 26006203 10.5152/tjg.2015.8001

[21] Ozturhan H Emekdaş G Sezgin O Seroepidemiology of *Fasciola hepatica* in Mersin province and surrounding towns and the role of family history of the Fascioliasis in the transmission of the parasite. Turk J Gastroenterol. 2009; 20(3):198–203. 19821202 10.4318/tjg.2009.0007

[22] Martínez-Barbabosa I Gutiérrez-Quiroz M Romero-Cabello R Seroepidemiology of fascioliasis in school children in Mexico City. Rev Biomed. 2006; 17:251–7.

[23] González LC Esteban JG Bargues MD Hyperendemic human fascioliasis in Andean valleys: An altitudinal transect analysis in children of Cajamarca province, Peru. Acta Trop. 2011; 120(1–2):119–29. 21767521 10.1016/j.actatropica.2011.07.002

[24] Esteban J-g Gonzalez C Curtale F Hyperendemic fascioliasis associated with schistosomiasis in villages in the Nile Delta of Egypt. Am J Trop Med Hyg. 2003; 69(4):429–37. 14640504

[25] Esteban J-G Flores A Angles R High endemicity of human fascioliasis between Lake Titicaca and La Paz valley, Bolivia. Trans R Soc Trop Med Hyg. 1999; 93(2):151–6. 10450437 10.1016/s0035-9203(99)90289-4

[26] Moghaddam A Massoud J Mahmoodi M Human and animal fascioliasis in Mazandaran province, northern Iran. Parasitol Res. 2004; 94(1):61–9. 15338292 10.1007/s00436-004-1169-6

[27] Ashrafi K Valero M Forghan-Parast K Potential Transmission of Human Fascioliasis Through Traditional Local Foods, in Northern Iran. Iran J Public Health. 2006; 35(2): 49–56.

